# Unraveling the Impact of Six Pentacyclic Triterpenes Regulating Metabolic Pathways on Lung Carcinoma Cells

**DOI:** 10.3390/ph17060694

**Published:** 2024-05-28

**Authors:** Anamaris Torres-Sanchez, Grace Torres, Sthephanie Estrada, Daraishka Perez, Carlos Garcia, Melissa Milian, Eddian Velazquez, Valerie Molina, Yamixa Delgado

**Affiliations:** 1 Biology Department, University of Puerto Rico-Rio Piedras, San Juan, PR 00931, USA; anamaris.torres@upr.edu; 2 Biochemistry Department, University of Puerto Rico-Medical Sciences Campus, San Juan, PR 00936, USA; 3 Biochemistry & Pharmacology Department, San Juan Bautista School of Medicine, Caguas, PR 00726, USAdaraishkapc@sanjuanbautista.edu (D.P.); eddianva@sanjuanbautista.edu (E.V.); valeriemp@sanjuanbautista.edu (V.M.); 4 Biology Department, University of Puerto Rico-Cayey, Cayey, PR 00736, USA; sthephanie.estrada@upr.edu; 5 Medical Program, Ponce Health Science University, Ponce, PR 00716, USA

**Keywords:** pentacyclic triterpenes, non-small cell lung carcinoma, phytochemicals, chemoresistance

## Abstract

Recently, there has been great interest in plant-derived compounds known as phytochemicals. The pentacyclic oleanane-, ursane-, and lupane-type triterpenes are phytochemicals that exert significant activity against diseases like cancer. Lung cancer is the leading cause of cancer-related death worldwide. Although chemotherapy is the treatment of choice for lung cancer, its effectiveness is hampered by the dose-limiting toxic effects and chemoresistance. Herein, we investigated six pentacyclic triterpenes, oleanolic acid, ursolic acid, asiatic acid, betulinic acid, betulin, and lupeol, on NSCLC A549 cells. These triterpenes have several structural variations that can influence the activation/inactivation of key cellular pathways. From our results, we determined that most of these triterpenes induced apoptosis, S-phase and G2/M-phase cycle arrest, the downregulation of ribonucleotide reductase (RR), reactive oxygen species, and caspase 3 activation. For chemoresistance markers, we found that most triterpenes downregulated the expression of MAPK/PI3K, STAT3, and PDL1. In contrast, UrA and AsA also induced DNA damage and autophagy. Then, we theoretically determined other possible molecular targets of these triterpenes using the online database ChEMBL. The results showed that even slight structural changes in these triterpenes can influence the cellular response. This study opens up promising perspectives for further research on the pharmaceutical role of phytochemical triterpenoids.

## 1. Introduction

Cancer is a leading cause of death around the world, where lung cancer is the most common cause of cancer-related deaths. Approximately 85% of all lung cancers are non-small cell lung carcinoma (NSCLC) [1]. Unfortunately, existing therapies do not cure most NSCLC patients. Even chemotherapy (e.g., platinum-based drugs such as cisplatin (CisPt)), the preferred and most effective treatment for lung cancer, only shows a complete response in 30% of individuals with NSCLC [2]. Additionally, chemoresistance, which results in more aggressive cancer cells, limits the efficacy of chemotherapy. Approximately 10–15% of patients receiving CisPt will relapse and invariably develop chemoresistance, with an expected survival time of no longer than a year [3]. In the last decade, the approvals for novel targeted and immunogenic anticancer agents have increased exponentially. From 2020 to 2023, sixteen of these new drugs were approved for the treatment of lung cancer, e.g., nivolumab (Opdivo^®^) and ipilimumab (Yervoy^®^) combined with a platinum-based drug is the first-line therapy for PDL1+ NSCLC [4]. For these reasons, new and adjuvant therapies are still needed. Phytochemicals such as triterpenes have demonstrated great potential against different cancers [5] and could work to overcome drug resistance mechanisms [6].

**Figure 1:**
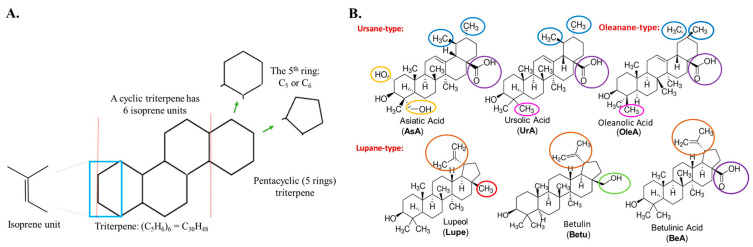
Structural representation of triterpenes. (**A**) Basic structure of a pentacyclic triterpene; (**B**) six pentacyclic triterpenes with their key substituents highlighted in color.

Triterpenes are secondary metabolites abundantly found in numerous plant species and composed of six isoprene units, resulting in a total of 30 carbon atoms in their molecular structure (see Figure 1A). Among the various triterpenes, pentacyclic triterpenoids exhibit approximately 200 different skeletons, showcasing remarkable structural diversity and potent biomedical activity [7]. Pentacyclic triterpenes that possess either five six-carbon (C_6_) rings (C 6-6-6-6-6) or four six-carbon (C_6_) rings and one five-carbon (C_5_) ring (C 6-6-6-6-5) are considered the most bioactive triterpenoids [8].

The ursane, lupane, and oleanane types are widely distributed within higher plants, including edible species. Within the ursane and oleanane types, asiatic acid (AsA), ursolic acid (UrA), and oleanolic acid (OleA) present a C 6-6-6-6-6 structural arrangement. These compounds exhibit very similar structures, with only slight variations in the substituent groups of the fifth C_6_ ring. For instance, AsA possesses an additional hydroxyl group at position 2 and a hydroxymethyl substituent at position 4. On the other hand, OleA differs from UrA in that a methyl substituent has been moved from position 19 to position 20. Interestingly, the lupane-type pentacyclic triterpenes, namely lupeol (Lupe), betulin (Betu), and betulinic acid (BeA), exhibit a C 6-6-6-6-5 structural arrangement. The primary distinction among these three compounds lies in the substituent group at position 17. Specifically, Lupe possesses a methyl group, Betu contains a hydroxymethyl group, and BeA features a carboxylic acid group at this position. These subtle but specific variations among these six pentacyclic triterpenoids may play a crucial role in their activity across metabolic pathways, especially in cancer [9]. Figure 1B provides a visual representation highlighting the differences between these pentacyclic triterpenes.

Herein, we investigated a library of six structurally similar pentacyclic triterpenes, AsA, UrA, OleA, Lupe, Betu, and BeA, against NSCLC. To study their cellular mechanistic effects, we determined the impact on the cell cycle, DNA, production of reactive oxygen (ROS), and activation of apoptosis and autophagy. In addition, we determined the protein expression of the mitogen-activated protein kinase/phosphatidylinositol 3-kinase (MAPK/PI3K) and the gene expression of ribonucleotide reductase (RR), programmed death-ligand 1 (PDL1), and signal transducer and activator of transcription 3 (STAT3), key markers of chemoresistance in NSCLC. This study opens up promising perspectives for further research on the role of triterpenoids as therapeutics, which ultimately will contribute to their more rational application in lung cancer therapy.

## 2. Results

### 2.1. Cytotoxicity and Therapeutic Index (TI)

**Figure 2:**
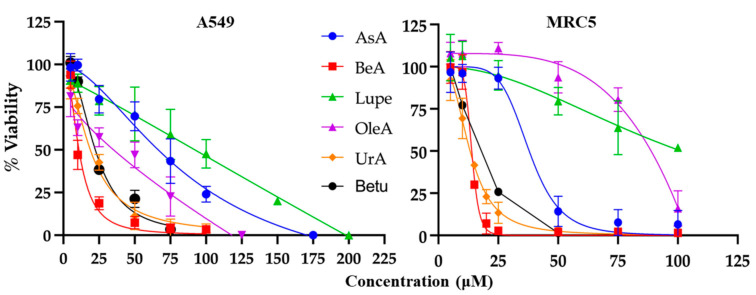
Cell death assays. MTS/PMS viability. Triterpenes were incubated for 24 h in A549 and MRC5 cells. Data show the average of eight measurements for each treatment (mean ± SD) in at least three independent experiments. All triterpenes were cytotoxic to A549 and MRC5 cells at a micromolar range. Both graphs show normalized data.

The MTS/PMS colorimetric assay was used to determine the viability of cells exposed to the triterpenes, generating a dose–response curve for NSCLC A549 cells and normal lung fibroblast MRC5 cells. Each triterpene was incubated at several μM concentrations for 24 h to determine the IC_50_. As expected, the cell viability decreased at increasing drug concentrations (Figure 2). The IC_50_ values were calculated using the data from Figure 2 and are summarized in Table 1. UrA was the most cytotoxic triterpene in MRC5 cells, and BeA was the most cytotoxic in the A549 cell line. In contrast, Lupe was the least cytotoxic in both cell lines.

The therapeutic index (TI) is a measurement of selectivity, comparing the IC_50_ of a drug against non-cancerous vs. cancerous cells. The OleA showed the highest TI (=2), while UrA showed the lowest TI (=0.57). Interestingly, Lupe and BeA had the same TI but BeA had the lowest IC_50_ for both cell lines.

### 2.2. Cell Cycle Arrest, DNA Damage, and Ribonucleotide Reductase (RR) Expression

**Figure 3:**
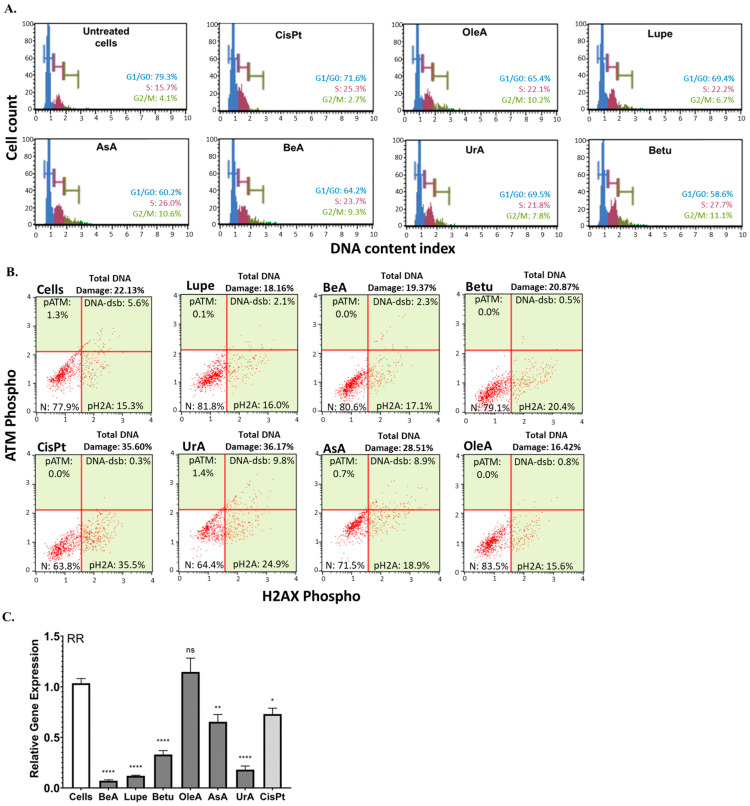
DNA-related pathways. (**A**). Cell cycle arrest assay. All pentacyclic triterpenes induced cell cycle arrest at the S phase and G2/M. (**B**). DNA damage assay. Only UrA and AsA induced the activation of the DNA damage machinery. (**C**). Gene expression of RR. Each assay was run in at least two independent experiments and samples were analyzed in triplicate. The statistical significance for *p* values was defined as **** < 0.0001, ** from 0.001 to 0.01, * from 0.01 to 0.05, and non-significant (ns) ≥ 0.05.

To characterize the effects of these triterpenes on nuclear processes, we evaluated the cell cycle populations and DNA damage machinery. Figure 3A demonstrates that all pentacyclic triterpenes produced cell cycle arrest in A549 cells at the S phase and G2/M phase in comparison to the negative control (untreated cells) and greater cycle arrest in G2/M in comparison to CisPt. In contrast, only UrA and AsA caused the activation of the DNA damage machinery compared to the negative control (untreated cells), as shown in Figure 3B. UrA was demonstrated to induce a similar DNA damage extent to CisPt chemotherapy. After we found these nuclear effects, we decided to test whether the expression of RR was also affected. Figure 3C shows the significant downregulation of the RRM1 gene compared to untreated cells, being more potent than CisPt. Only OleA did not downregulate RR.

### 2.3. Apoptosis: Externalization of PS and Caspase 3 Activity

**Figure 4:**
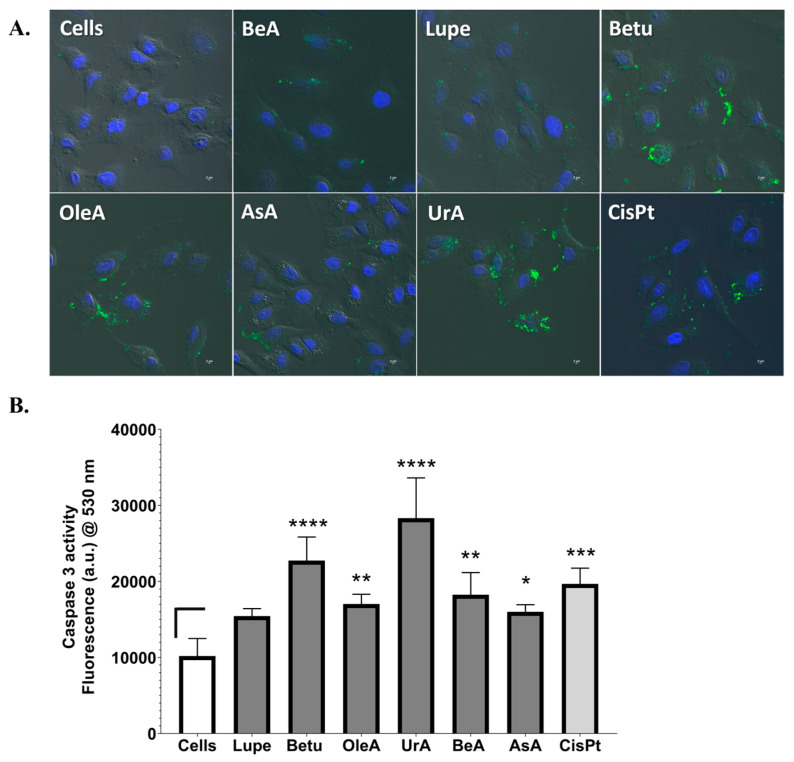
Apoptotic-related pathways. (**A**). Externalization of phosphatidylserine. Triterpenes were incubated for 24 h in A549 and then with Annexin V (green) and NucBlue DAPI (blue); scale bars are shown (2 μm). The brightfield, green, and blue fluorescence channels are overlaid in these images. All triterpenes showed the activation of early apoptosis to A549. Each sample was analyzed in duplicate in two independent experiments. The images shown were selected arbitrarily. All images were taken at 20× magnification. (**B**). Caspase 3 activity assay. All triterpenes activated caspase 3, except Lupe. UrA and Betu showed the highest activation. Data shown as mean ± SD in three independent experiments and samples were analyzed in triplicate. The statistical significance for p values was defined as **** < 0.0001, *** from 0.001 to 0.0001, ** from 0.001 to 0.01, * from 0.01 to 0.05, and non-significant (ns) ≥ 0.05.

Given the importance of the activation apoptotic processes to eradicate cancer cells, we chose to determine the markers of early apoptosis and intrinsic apoptosis pathways after 24 h in NSCLC cells. We used confocal microscopy to identify the activation of the apoptotic machinery by the externalization of PS on the cell membrane using Annexin V dye and NucBlue DAPI as the nuclear markers. In Figure 4A, we demonstrate that all triterpenes showed early apoptosis activity (green marker), similarly to CisPt. The complete set of images for each individual channel can be found in the Supporting Information (Figure S1). Qualitatively, we observed that UrA and Betu showed the strongest activation and Lupe and BeA the lowest.

Figure 4B shows that all triterpenes activated the caspase 3 enzymatic machinery during a 24 h treatment period, except for Lupe, compared to the negative control (untreated cells). However, UrA and Betu produced higher caspase 3 activation than CisPt, where UrA was the highest.

### 2.4. Total ROS and Mitochondrial ROS Production and Autophagy

**Figure 5:**
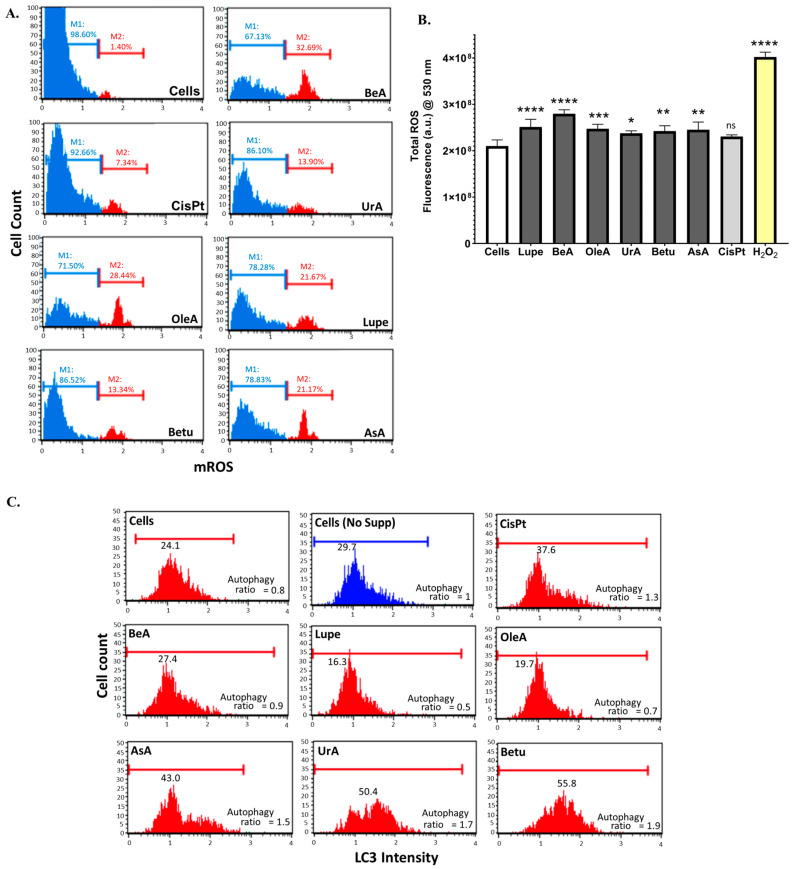
Oxidative stress-related processes. (**A**). Mitochondrial ROS production assay. Dihydroethidium (DHE) dye reacts with the superoxide species mostly produced by mitochondrial damage. All six triterpenes induced the production of mROS. Each assay was run in at least two independent experiments and samples were analyzed in triplicate in the instrument. (**B**). Total ROS production assay. DCF dye reacts with the peroxide (H_2_O_2_) species as the final product during oxidation in cells. All six triterpenes induced the production of ROS. Data shown as mean ± SD in three independent experiments and samples were analyzed in triplicate. (**C**). Autophagy induction assay. For this experiment, we included a positive control (No supp) by removing the serum from the culturing medium (starvation) of the untreated cells. Betu, AsA, and UrA activated the autophagic response. Each assay was run at least in two independent experiments and samples were analyzed in triplicate in the instrument. The statistical significance for p values was defined as **** < 0.0001, *** from 0.001 to 0.0001, ** from 0.001 to 0.01, * from 0.01 to 0.05, and non-significant (ns) ≥ 0.05.

Oxidative stress, as ROS, plays an interesting role by regulating cell death mechanisms such as autophagy [8]. Thus, we determined the effect of these triterpenes on this process. Figure 5A shows that all triterpenes increased the superoxide ROS generated from mitochondria compared with the negative control, and some of their levels were higher than that of CisPt. BeA caused the largest level of mROS, whereas the lowest was produced by AsA. Then, we measured the total amount of ROS to confirm our results for mROS.

Figure 5B showed a very similar tendency in the mROS results. All triterpenes induced an increase in the total ROS from peroxide species. In the case of CisPt, the increase in the total ROS was not statistically significant. For this assay, we used cells incubated with 3 μM of H_2_O_2_ as a positive control.

Figure 5C shows that Betu, AsA, and UrA induced an autophagic response similar to that of CisPt, compared to the controls (negative control untreated cells, ratio = 0.8, and positive control untreated starving cells, ratio = 1). BeA had a similar ratio to the untreated cells, while Lupe and OleA were shown to inhibit the LC3-II activity.

### 2.5. MAPK/PI3K Protein Expression and PDL1 and STAT3 Gene Expression

**Figure 6:**
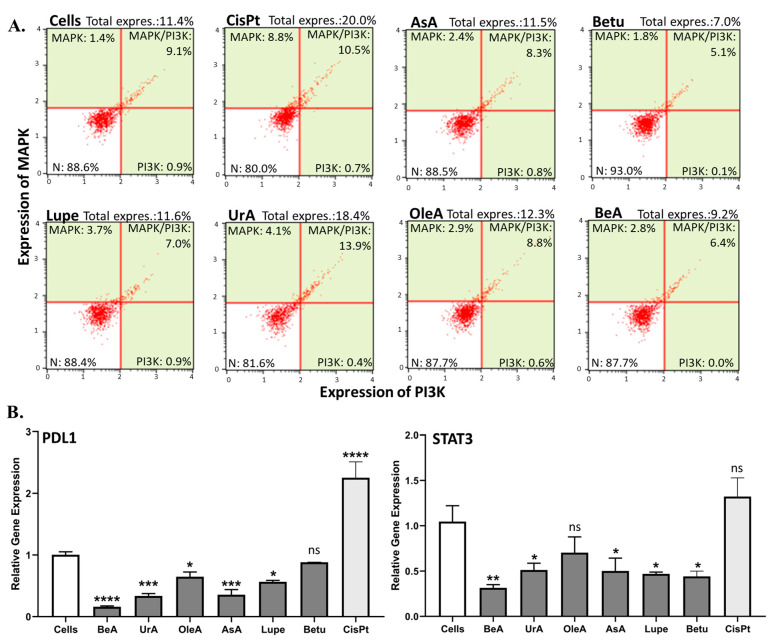
Protein and gene expression assays. (**A**). MAPK/PI3K protein expression. Only UrA increased and Betu diminished MAPK/PI3K expression. Each assay was run at least in two independent experiments and samples were analyzed in triplicate in the instrument. (**B**). PDL1 and STAT3 gene expression using real-time qPCR. Only OleA and Betu did not inhibit the STAT3 and PDL1 genes, respectively. The data were analyzed in triplicate and are expressed as the mean ± SD of three independent experiments. The statistical significance for p values was defined as **** < 0.0001, *** from 0.001 to 0.0001, ** from 0.001 to 0.01, * from 0.01 to 0.05, and non-significant (ns) ≥ 0.05.

The MAPK, PI3K, PDL1, and STAT3 pathways are commonly studied in cancer due to their involvement in several cellular processes, especially chemoresistance [9]. These were assessed in this study in A549 cells after 24 h incubation with the triterpenes. Figure 6A shows that UrA was the only one that induced the simultaneous expression of MAPK and PI3K, similarly to CisPt, while Betu could slightly inhibit the total expression of MAPK/PI3K, compared to untreated cells. In contrast, BeA, AsA, Lupe, and OleA did not affect the expression of MAPK/PI3K.

In the RT-qPCR gene expression assays (Figure 6B), most triterpenes significantly inhibited PDL1 and STAT3. OleA and Betu, on the other hand, did not inhibit the STAT3 and PDL1 genes, respectively. The lupane-type BeA is superior in suppressing both genes.

### 2.6. Theoretical Predictions of Molecular Targets

Theoretical analysis not only enriches our understanding of the pharmacokinetic properties of drugs, but also provides insights into possible protein targets, thereby increasing the robustness of the experimental results. Based on the results of our experiments, we decided to incorporate a theoretical prediction analysis of possible molecular targets using the comprehensive ChEMBL database.

From the results of this theoretical analysis of 339 biological targets, the program predicted more than 20 targets for each triterpene, with different confidence levels. We selected only those targets that met the three confidence levels (70, 80, 90%) and had the strongest activity thresholds (5–6 numerical values). The whole set of targets (~10) that fulfilled the strict criteria are shown in the Supporting Information, Table S1. From this whole set, we summarize the targets related to cancer metabolism, chemoresistance, and metastatic processes in Table 2. We use different colors to match the same targets across the different triterpenes. ChEMBL only predicts the interactions of the two molecules in the comparison and not the activation or inhibition of these targets per se.

### 2.7. Results Summary

Table 3 summarizes the findings from all of the experimental mechanistic pathways studied in this project. The lupane-type BeA is the pentacyclic triterpene that affects most of the key pathways with a lower concentration (lowest IC_50_) and is the most potent in the production of total and mROS and the downregulation of STAT3 and PDL1 in the NSCLC A549 cells. These results indicate that the structure of the triterpene affects its interaction with different pathways.

## 3. Discussion

**Figure 7:**
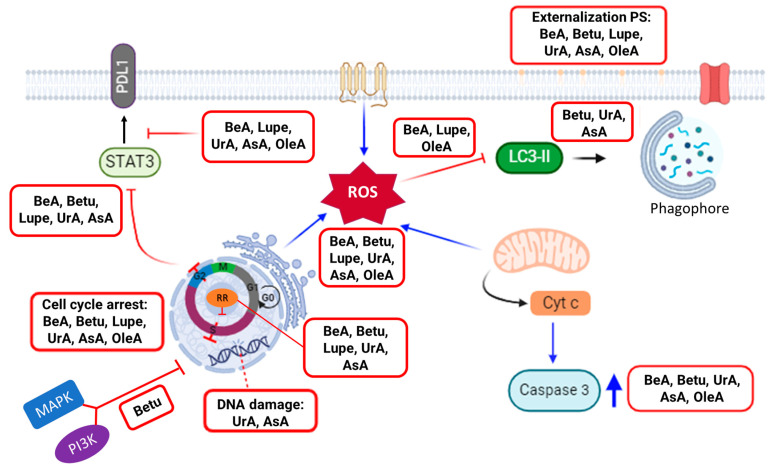
Summary of the experimental pathways’ relationships as activated or inhibited by the six pentacyclic triterpenes against NSCLC.

Triterpenes are known for their wide range of benefits, including their anti-inflammatory, antimicrobial, antiviral, analgesic, anti-tumorigenic, and immunomodulatory characteristics [5,6,7,8,11]. For this project, we aimed to study a library of six pentacyclic triterpenes with similar structures to determine which one was the most potent against lung carcinoma, one of the deadliest cancers. While previous research has demonstrated the potency of these triterpenes, our study aimed to determine which one had the most effective structural arrangement for potency, selectivity, and the targeting of key pathways against chemoresistance, the major problem contributing to chemotherapy failure. We selected pentacyclic triterpenes because, in our previous study, we found that the fifth ring of the structure is essential for the cytotoxic activity in NSCLC A549 cells. For instance, even at the highest concentration of 100 μM, β-sitosterol and cholesterol (tetracyclic triterpenes) did not kill A549 cells [10]. Thus, this study explored the underlying mechanistic principles (Figure 7) with a particular emphasis on key pathways to induce cell death (i.e., cell cycle arrest, expression of RR, DNA damage, activation of caspase 3, and oxidative stress) but also in chemoresistance-related processes (i.e., inhibition of autophagy; MAPK, PI3K, STAT3, and PDL1 expression).

Our experimental results revealed distinct patterns in the response of NSCLC cells to the six pentacyclic triterpenes under scrutiny. The viability assays demonstrated that Lupe had the highest IC_50_, OleA had the best TI, and BeA had the lowest IC_50_ with a TI >1. Normal lung cells such as MRC5 have been considered as cancer-associated fibroblasts (CAF), even when they are non-cancerous cells [12]. This means that an effective therapy must kill both cell lines (NSCLC and CAF) but behave aggressively towards cancerous cells. In this context, BeA showed the lowest IC_50_ to kill both cell lines (NSCLC and CAF) but behaved more aggressively towards the NSCLC A549 cells among the six triterpenes. These results suggest that a methyl substituent in lupane-type triterpenes, instead of carboxylic acid or hydroxyl, can drastically diminish their cytotoxicity. On the other hand, changes in the methyl substituents in OleA (vs. UrA) diminished their cytotoxicity in cells but increased their selectivity. Previous studies have found similar IC_50_ values in the μM range in a 24 h treatment period for the six selected pentacyclic triterpenes exposed to different cancer cell types [13,14,15,16,17,18]. This highlights the versatility of using triterpenes against cancer in different organs and tissues.

Nuclear processes such as the cell cycle are important pathways that promote cancer progression. Cell cycle arrest in response to DNA damage is a critical mechanism that helps to prevent the propagation of cancer cells and to induce apoptosis [19]. In this study, all triterpenes produced cell cycle arrest in the S phase and G2/M phase. There are several studies that have shown similar results, where these triterpenes exhibited S-phase and G2/M-phase arrest in myeloma, gastric, breast, and lung cancer cells [20,21,22,23]. These results suggest that, regardless of their structural variations, these six pentacyclic triterpenes may trigger cell cycle arrest in these two critical divisions in NSCLC, implying that they all may be able to prevent the proliferation of these cancer cells. In addition, only the ursane-type UrA and AsA caused DNA damage, implying their other potential targets to kill cancer cells compared to the other four triterpenes. These results revealed that, unlike lupane-type triterpenes, ursane-type triterpenes might employ DNA damage machinery to induce apoptosis. These results support previous studies that highlight UrA’s ability to increase DNA damage and chemosensitize A549 cells [24]. In contrast, there are several studies that have demonstrated that lupane-type triterpenes are able to cause DNA damage [25,26]. RR is an enzyme complex composed of two subunits: a catalytic alpha subunit (RRM1) and a radical-generating beta subunit (RRM2). These subunits are responsible for catalyzing the rate-limiting step for DNA synthesis and are known to be elevated in a variety of cancers because of their influence on invasion, including NSCLC [27]. This study found that the lupane- and ursane-type triterpenes downregulated the RRM1 subunit gene. No previous studies have demonstrated that these pentacyclic triterpenes affect RR. However, the literature indicates that another pentacyclic (C6-6-6-6-6) triterpene, celastrol, downregulates RRM2 [28].

Another important pathway is the activation of apoptosis through the mitochondrial response and the externalization of PS on the cell membrane. The disruption of the mitochondrial membrane potential can lead to the release of cytochrome c, which activates caspase 3, initiating intrinsic apoptosis and, then, cell death [29]. Another explanation for this mechanism is that the proapoptotic protein Bid can directly activate caspase 8 or translocate to the mitochondrion, leading to the release of cytochrome c to activate caspases 3/7, regardless of the mitochondrial potential [30]. Most studies in the literature show that pentacyclic triterpenes induce both intrinsic and extrinsic apoptosis by caspase 3 and caspase 8, respectively [31]. In the current study, all triterpenes except Lupe activated caspase 3. In addition, all triterpenes activated the early apoptotic cellular process on the A549 cells, where Lupe and BeA induced the lowest level of activation. Interestingly, BeA demonstrated the low activation of early apoptosis and caspase 3 and no DNA damage, while UrA showed the activation of the DNA damage machinery, as well as the strongest activation of caspase 3 and the externalization of PS. Structurally, BeA and UrA were demonstrated to more aggressively attack different pathways in NSCLC.

Oxidative stress, such as ROS levels, can modulate autophagy. Mild amounts of ROS promote the autophagic response, while high concentrations inhibit autophagy and promote cell death [32]. To determine the mROS, we used the DHE dye, which reacts mainly with superoxide species [33]. Although all triterpenes produced mROS, compared to the untreated cells, the amounts of mROS from BeA, OleA, and Lupe were, on average, 26.2%, while Betu, AsA, and UrA showed an average of 10.1%. Thus, BeA, OleA, and Lupe did not activate autophagy because of the high concentration of ROS. On the other hand, Betu, AsA, and UrA activated the autophagic response via the lower levels of ROS. Although mitochondria are the main suppliers of ROS, other organelles, such as the endoplasmic reticulum, also play important roles in ROS production [34]. Thus, we decided to test the total amount of ROS using the DCF dye, which labels mostly the peroxide species as the end products of free radicals during oxidation reactions. Again, BeA showed the highest increase in total ROS, in agreement with the increase in mROS. Numerous studies agree with our results showing that triterpenes can induce cell death via the production of ROS, as summarized in the review in [31]. It is important to understand that autophagy can suppress tumor development in the early stages, but, once the tumor is established, it promotes chemotherapy resistance [35]. Based on the structural aspects of these triterpenes, our results suggest that the skeletal carbon ring structure of the pentacyclic triterpenes is essential in triggering the formation of mROS. Moreover, the results revealed that most of the differences in the six pentacyclic triterpenes’ structures and their substituents changed the ROS production levels, but this was not sufficient to inhibit this process. A possible explanation is that these triterpenes inactivate iron complex I and iron complex III in the electron transport chain. Thus, under increased ROS levels, iron is released from these complexes and apoptosis is switched to a process called ferroptosis, which depends on the presence of free iron and high oxidant conditions [36]. In this way, BeA can activate apoptosis but mostly ferroptosis by high ROS and no autophagy. In contrast, UrA can activate mostly apoptosis and autophagy by DNA damage and moderate levels of ROS.

MAPK/PI3K has been linked to the upregulation of PDL1, which leads to immune suppression and chemoresistance [9,37]. Meanwhile, the STAT3-mediated upregulation of PDL1 expression in cancer cells has been observed [37]. In our experiments, only UrA slightly increased the expression of MAPK/PI3K when compared to the negative (untreated cells) and positive (CisPt) controls. These results suggest that lupane-type triterpenes with a 6-6-6-6-5 structural arrangement (where Betu < BeA < Lupe) are superior in limiting cell progression and survival by MAPK/PI3K. Regarding the chemoresistance markers STAT3 and PDL1, our results revealed that most of the pentacyclic triterpenes suppressed these two genes, where the chemotherapy CisPt clearly upregulated PDL1. However, BeA was the greater inhibitor of PDL1 and STAT3 gene expression, while it did not increase MAPK/PI3K expression. This outcome is also in agreement with our hypothesis of the activation of ferroptosis, which may sensitize chemoresistant cancer cells [36]. In the literature, we found similar results for MAPK, PI3K, PDL1, and STAT3 after BeA treatment [38,39]. UrA was also shown to downregulate STAT3 and PDL1 [40].

Computational approaches provide detailed information to predict the pharmacokinetic properties, biological activity, and molecular mechanisms of small molecules, including their interactions with specific protein targets with associated signaling pathways [41]. For example, from a database of 339 proteins, ChEMBL predicted interactions with approximately 25 targets with an activity threshold of 5–7.5. In evaluating the data, we selected targets with stronger predictive properties: lower activity threshold values and confidence intervals of 70, 80, and 90%. These protein targets were of the following types: (a) membrane-associated proteins, receptors, and ion channels; (b) nuclear proteins; (c) cytoplasmic enzymes; and (d) cytokines and signaling molecules (shown in Table S1). Thus, Table 2 shows only the cancer-related targets (~five for each triterpene). Most of these triterpenes have very similar targets. Interleukin-2 is a common target of all six triterpenes. Dihydrofolate reductase and WD repeat-containing protein 5 are targeted by all except AsA. TNF-alpha is targeted by all except Lupe and Betu. The LSD1/CoREST complex is targeted by all except OleA and UrA. Adenosine deaminase is only targeted by lupane-type triterpenes. On the other hand, mucosa-associated lymphoid tissue lymphoma translocation protein 1 (MALT1) and gamma-secretase are only targeted by AsA, and carbonic anhydrase IX is only targeted by Lupe. Knowing that ChEMBL calculates the probability of each triterpene interacting with the protein targets in the database (not their specific activation or inhibition), we can make several important connections based on the molecules predicted to be targeted. The most important findings from these predictions are that these pentacyclic triterpenes have the ability to trigger an inflammatory response (IL-2 and TNF-alpha) by the activation of adenosine deaminase. This process may induce immunostimulation to attack tumor cells [42]. In addition, only AsA could target MALT1, which acts as a scaffolding protein to drive immune activation [43]. This prediction confirmed our experimental results on the downregulation of PDL1 and STAT3. Furthermore, these triterpenes can interact with proteins such as the LSD1/CoREST complex, WD repeat-containing protein 5, and dihydrofolate reductase, which regulate the cell cycle, DNA synthesis, and genomic instability [19]. This prediction could explain our experimental results regarding cell cycle arrest in the S phase and G2/M. Similarly, targeting carbonic anhydrase IX could disrupt the stability of the hypoxic tumor environment. Targeting carbonic anhydrase IX is a potential therapeutic strategy to starve tumors and limit their spread [44]. In particular, AsA is predicted to interact with gamma-secretase, which is a novel therapeutic strategy in cancer that involves blocking Notch cleavage [45].

In summary, BeA emerges as the triterpene exerting the most widespread influence on several key pathways related to ROS-mediated cell death, with the lowest IC_50_ and a TI > 1, and inhibits the chemoresistance-related genes STAT3 and PDL1. Theoretically, BeA can also modulate the immune response by interacting with IL-2, TNF-alpha, and adenosine deaminase. Based on the structure, the C 6-6-6-6-5 arrangement substituted with a carboxylic acid and a HC=C-CH_2_ group is the most potent against NSCLC. These findings collectively indicate that triterpenes impact different pathways depending on the substituents of their rings. While BeA has the lower IC_50_ of 11 μM, it is important to note that its TI is narrow but above 1. However, OleA showed the best TI (= 2), making it a promising candidate for derivatization. A low IC_50_ reflects the potency of a drug in inhibiting cancer cell growth, while a high TI is crucial in ensuring the safety and tolerability of the drug. Both parameters are important considerations in cancer therapeutics, with the ideal drug having both a low IC_50_ and a high TI. However, MEK inhibitors, clinically approved chemotherapeutics, have a TI ~ 1 [46]. Based on these findings, future research can utilize the most effective patterns of parent molecules BeA and OleA for derivatization. Specifically, the alternate methyl groups of the fifth C_6_ ring of OleA could be the key to higher selectivity. A possibility to consider is a BeA derivative that includes the original substituent of the HC=C-CH_2_ group in the fifth C_5_ ring to maintain the potency, as well as the alternate methyl groups of OleA to increase the selectivity. To validate our arguments about OleA, two synthetic derivatives of OleA, CDDO-Me and CDDO-Im, were studied in phase I clinical trials [47,48]. Furthermore, OleA in combination with curcumin was examined in a phase I clinical trial [49]. On the other hand, selectivity of BeA could be increased by its encapsulation into a targeted drug delivery system (DDS). Thus, we previously loaded BeA in a protein-based DDS, demonstrating that BeA was synergized with doxorubicin chemotherapy in A549 cells [50].

## 4. Materials and Methods

### 4.1. Chemicals and Reagents

Aqueous solutions were prepared with sterile (autoclave conditions: 121 °C and 18 PSI), high-quality nanopure water (18.2 MΩ cm resistivity, Thermo Scientific™ Barnstead™ Easypure™ II system (Thermo Fisher Scientific, Waltham, MA, USA)).

Asiatic acid, ursolic acid, oleanolic acid, betulin, lupeol, betulinic acid, 2′,7′-dichlorodihydrofluorescein diacetate (DCF) and cis-diamminedichloroplatinum (II) (cisplatin, CisPt) were purchased from Sigma-Aldrich (St. Louis, MO, USA). The cell lines A549 (human lung adenocarcinoma; ATCC CCL-185) and MRC5 (human fibroblast-like cells; ATCC CCL-171) were obtained from the American Type Culture Collection (Manassas, VA, USA). The CellTiter 96 Aqueous Non-Radioactive Cell Proliferation Assay (3-(4,5-dimethylthiazol-2-yl)-5-(3-carboxymethoxyphenyl)-2-(4-sulfophenyl)-2H-tetrazolium (MTS) reagent) was obtained from the Promega Corporation (Madison, WI, USA). The cell cycle, MAPK/PI3K expression, DNA damage, and mitochondrial oxidative stress assays were obtained from the Luminex Corporation (Austin, TX, USA). Reagents for Taqman gene expression assays were purchased from Applied Biosystems (Waltham, MA, USA). All other chemicals were purchased from various suppliers, of analytical grade, and used without further purification.

### 4.2. Cell Culture Conditions

The human NSCLC A549 and normal lung MRC5 cell lines were maintained following the ATCC protocols. These cells were cultured in Dulbecco’s Modified Eagle’s Medium (DMEM-high glucose) containing 1% L-glutamine, 10% fetal bovine serum (FBS), and 1% penicillin/streptomycin. Cells were kept in a humidified incubator under 5% CO_2_ and 95% air at 37 °C. We conducted all of the experiments before the cells reached 25 passages. For all experiments, the triterpenes were initially dissolved in pure dimethylformamide (DMF) and subsequently diluted in phosphate-buffered saline (PBS), ensuring a final DMF concentration in the cells of less than 1%. CisPt (25 μM) was included as a control to compare the effects of the triterpenes with the most used chemotherapy in NSCLC.

### 4.3. Cell Viability

The 50% inhibitory concentration (IC_50_) of some of these triterpenes in A549 NSCLC cells were previously determined by us [10]. A similar procedure was performed to determine the IC_50_ of the triterpenes in a normal lung fibroblast cell line (MRC5). In brief, the A549 and MRC5 cells were seeded into 96-well plates at a density of 1 × 10^5^ cells/mL in supplemented DMEM medium. The cells were treated with specific concentrations (A549: 5, 10, 25, 50, 75, 100, 125,150, 175, 200 μM; MRC5: 5, 10, 25, 50, 75, 100 μM) of the triterpenes and incubated for 24 h. Then, 10 uL of the MTS reagent from the CellTiter 96^®^ Aqueous Non-Radioactive Cell Proliferation Assay (Promega G5421) was added to each well. Then, we incubated the plates at 37 °C and in a 5% CO_2_ atmosphere for 1 h. After incubation, the absorbance was measured at 492 nm (*n* = 8) in a microplate reader spectrophotometer (Thermoscientific Multiskan FC). The cell viability was calculated as follows:

% Viability = (sample absorbance − sample media absorbance)/(untreated cells’ absorbance − untreated cells’ media absorbance) × 100

The cell viability data were presented by plotting the values with an average of eight measurements for each treatment (mean ± SD) in at least three independent experiments. The IC_50_ values were calculated with the GraphPad Prism 9 software (Prism 9; GraphPad by Dotmatics, San Diego, CA, USA) using the dose–response curve. The data were normalized by the non-linear fit of the log (drug inhibition) vs. normalized response variable slope to obtain the best fit IC_50_ and R-squared values.

### 4.4. Apoptosis Externalization of Phospatidylserine (PS) Annexin V Assay

Early apoptotic activity was evaluated by the externalization of PS using the Alexa Fluor^®^ 488 Annexin V Apoptosis Reagent (Molecular Probes™) and NucBlue ReadyProbes Reagent™ (Thermo Fisher Scientific). For this assay, A549 cells were seeded in 8-well cover slip plates with a density of 1 × 10^5^ cells/mL for 24 h in supplemented DMEM. Then, the A549 cells were incubated with the triterpenes (IC_25_) and controls for 24 h. Afterward, the cells were washed with 1× PBS and incubated with NucBlue (DAPI analog) and Annexin V for 15 min in the dark. The cells were fixed with 3.7% formaldehyde solution for 15 min, washed three times with 1× PBS, and covered with glycerol before visualization. The plates were observed by confocal microscopy using a Nikon Eclipse Ti microscope (Nikon Instruments Inc., El Segundo, CA, USA), utilizing a 20× oil objective with filter sets compatible with DAPI (Ex/Em = 405/460 nm) and Annexin V Alexa Fluor (Ex/Em = 488/525 nm). The fluorescence intensity was analyzed using the NIS-Elements Viewer program (version 5.21 64-bit) (Nikon Instruments Inc.). Each sample was analyzed in duplicate in two independent experiments. The fluorescence intensity was adjusted using untreated cells for each fluorescence channel (green and blue). Subsequently, this calibrated intensity was applied for the analysis of all treated cells.

### 4.5. Caspase 3 Activity Assay

The intrinsic apoptosis activity was evaluated by the quantification of caspase 3 enzymatic activity using the Apo-ONE^®^ Homogeneous Caspase-3/7 Assay (Promega G7792). A549 cells were seeded in a concentration of 1 × 10^5^ cells/mL into black 96-well plates with a clear bottom. After 24 h, the cells were incubated with the triterpenes (IC_50_) and controls for 24 h. After the completion of the incubation, 50 μL of Apo-ONE^®^ Caspase-3/7 Reagent (1:100 rhodamine 110 bis-(N-CBZ-l-aspartyl-l-glutamyl-l-valyl-aspartic acid amide) (Z-DEVD-R110) substrate diluted in lysis buffer) was added to each well. The plate was gently mixed using a plate shaker at 150 rpm for 2 h at room temperature. Then, the fluorescence of each well was measured (Ex/Em = 485/530 nm) in the Tecan^®^ M200 plate reader.

The caspase 3 activity experiments were performed in triplicate. All data were expressed by plotting the values with an average of three measurements for each treatment condition as the mean ± SD and analyzed with the software GraphPad Prism 9 (San Diego, CA, USA). Statistical analysis was performed using one-way analysis of variance (ANOVA) to compare the mean value of each type of treated cells versus the control (untreated cells). The statistical significance defined by the software for *p* values was **** < 0.0001, *** from 0.001 to 0.0001, ** from 0.001 to 0.01, * from 0.01 to 0.05, and non-significant (ns) ≥ 0.05.

### 4.6. Flow Cytometry Analysis

We used flow cytometry to analyze several cellular mechanistic pathways in A549 cells after incubation with the triterpenes. In general, the cells were seeded in a 6-well plate at a density of 1 × 10^6^ cells/mL and incubated for 24 h in supplemented DMEM. After 24 h, the A549 cells were treated with the triterpenes, following each manufacturer’s protocol. All of the following experiments were performed in a Muse^®^ Cell Analyzer (EMD Millipore Corporation, Temecula, CA). The instrument was calibrated before each assay using the Muse^®^ System Check Kit (Luminex MCH100101, Luminex Corporation). Each assay was run at least in two independent experiments. For each experiment, all samples were analyzed in triplicate in the instrument and the capillary line was washed after each sample run to avoid artifacts. In addition, to address the issue of cell doublets, we used the Muse^®^ Cell Dispersal Reagent (Luminex Corporation) in all experiments. The concentration of the triterpenes in each assay was selected depending on the cellular concentration necessary to generate the gain signal in the instrument. This means that we attempted to select concentrations avoiding excessive cytotoxicity to accurately determine the respective signal from each assay.

#### 4.6.1. Cell Cycle Arrest

A549 cells were incubated with the triterpenes (IC_25_) for 24 h. Then, the cells were scraped, centrifuged, and fixed using cold 70% ethanol. Afterward, the fixed cell pellet was incubated with the propidium iodide (PI)-based cell cycle reagent (Luminex MCH100106 kit, Luminex Corporation) for 30 min for immediate measurement.

#### 4.6.2. DNA Damage Induction

A549 cells were incubated with the triterpenes (IC_50_) for 24 h. Then, the A549 cells were scraped and centrifuged. The pellet was suspended in the assay buffer from the Luminex MCH200107 kit (Luminex Corporation). Later, the cell pellet was fixed and permeabilized. Afterward, the antibodies anti-pH2AX (histone) and anti-pATM (ataxia-telangiectasia mutated) were added and incubated for 30 min. Next, the cell pellet was washed and suspended in fresh assay buffer for measurement.

#### 4.6.3. Mitochondrial Oxidative Stress

The production of reactive oxygen species (ROS) by mitochondria was determined using the Oxidative Stress Kit (Luminex MCH100111, Luminex Corporation). After the A549 cells were treated with the triterpenes (IC_25_) for 24 h, the cells were scraped and centrifuged. For ROS, the cell pellet was incubated with dihydroethidium (DHE) dye for 1 h at 37 °C.

#### 4.6.4. Autophagy Activation

The Muse^®^ Autophagy LC3 Antibody-Based Kit (Luminex MCH200109, Luminex Corporation) was used to determine autophagy activation by monitoring lipidated LC3-II. After the A549 cells were treated with the triterpenes (IC_25_) for 24 h, autophagy reagent A was added for 4 h to prevent the lysosomal degradation of LC3-II. Then, the cells were detached and centrifuged. The pellet was suspended and incubated for 30 min in the autophagy reagent B containing anti-LC3-II mouse monoclonal antibody. Finally, the cell pellet was washed and suspended in fresh assay buffer for measurement. The autophagy response was calculated using the following formula:

Autophagy ratio = mean autophagy value of treated or untreated cells/mean autophagy value of starving cells.

The starving cells were labeled ‘No supp’ as non-supplemented cells. These served as the positive signal for autophagy and were designated a ratio of 1 since they were compared to themselves. A ratio ≥1 indicates the activation of the autophagic response.

#### 4.6.5. MAPK/PI3K Expression

The Muse^®^ P13K/MAPK Dual Pathway Activation Kit (Luminex MCH200108, Luminex Corporation) was used to determine MAPK/PI3K expression. After the incubation treatment with the triterpenes (IC_25_) for 24 h, the A549 cells were scraped, centrifuged, and washed once with 1× PBS. Next, the fixation buffer was incubated for 10 min and then the permeabilization buffer for another 10 min. Both incubations were performed on ice. Later, the antibody cocktail containing anti-phospho-Akt and anti-phospho-ERK1/2 was incubated for 30 min. Afterward, the cell pellet was washed and suspended in fresh assay buffer for measurement.

### 4.7. Total Oxidative Stress

The production of total ROS was determined using the 2′,7′-dichlorodihydrofluorescein diacetate (DCF) fluorescent dye. A549 cells were seeded in a concentration of 1 × 10^5^ cells/mL into black 96-well plates with a clear bottom. After 24 h, the cells were incubated with the triterpenes (IC_25_) and controls for 24 h. Then, 50 μL of DCF (10 μM) was added to each well and they were incubated for 1 h at room temperature. Then, the fluorescence of each well was measured (Ex/Em = 485/530 nm) in the Tecan^®^ M200 plate reader.

This experiment was performed in triplicate. All data were expressed by plotting the values as an average of three measurements for each treatment condition as the mean ± SD and analyzed with the software GraphPad Prism 9. Statistical analysis was performed using one-way analysis of variance (ANOVA) to compare the mean value of each treated type of cells versus the control (untreated cells). The statistical significance defined by the software for *p* values was **** < 0.0001, *** from 0.001 to 0.0001, ** from 0.001 to 0.01, * from 0.01 to 0.05, and non-significant (ns) ≥ 0.05.

### 4.8. RNA Extraction and Real-Time (RT) Quantitative PCR

A549 cells were seeded into 6-well plates at a density of 1 × 10^6^ cells/mL in supplemented DMEM. The cells were incubated with the triterpenes (IC_25_) for 24 h. Total RNA was extracted from the cells using the TRIzol reagent (Invitrogen Corporation, Carlsbad, CA, USA) according to the manufacturer’s instructions. The purity and concentration of RNA were assessed using a NanoDrop spectrophotometer (Thermo Fisher Scientific). cDNA was synthesized using the High-Capacity cDNA Reverse Transcription Kit (Applied Biosystems) according to the manufacturer’s instructions.

RT-qPCR was performed on a StepOnePlus^®^ Real-Time PCR System (Thermo Fisher Scientific) using TaqMan Gene Expression Assays (Applied Biosystems) for PDL1 (CD274; Assay ID: Hs01125297_m1), ribonucleotide reductase (RR) M1 (Assay ID: Hs01040697_m1), and STAT3 (Assay ID: Hs01047586_g1). Each reaction consisted of 10 ng of cDNA, TaqMan Gene Expression Master Mix (Applied Biosystems), and TaqMan probes. The PCR cycling conditions were as follows: 95 °C for 10 min, followed by 40 cycles of 95 °C for 15 s and 60 °C for 60 s. The relative gene expression levels were calculated using the ΔΔCt method. GAPDH (Assay ID: Hs02758991_g1) was used as the endogenous control for normalization.

All RT-qPCR experiments were performed at least in triplicate. All data were expressed by plotting values with an average of four measurements for each treatment condition as the mean ± SD and analyzed with the software GraphPad Prism 9. Statistical analysis was performed using one-way analysis of variance (ANOVA) to compare the mean value of each treated type of cells versus the control (untreated cells). The statistical significance defined by the software for *p* values was **** < 0.0001, *** from 0.001 to 0.0001, ** from 0.001 to 0.01, * from 0.01 to 0.05, and non-significant (ns) ≥ 0.05.

### 4.9. Theoretical Target Predictive Analysis

Computational analysis is an approach that helps to identify novel targets from extensive databases. We selected the curated database of bioactive molecules ChEMBL (from EMBL’s European Bioinformatics Institute) for our analysis. ChemBL was accessed through the online platform (https://www.ebi.ac.uk/chembl/) (accessed on 1 May 2024), where we searched the chemical structures and ChemIDs of the six pentacyclic triterpenes of interest. By applying filters such as human species specificity, active/inactive, confidence levels, and activity thresholds (logarithmic potency values), we retrieved the predictive information on the targets, including the protein names and triterpene compound IDs. The target prediction returned a result of ‘active’ or ‘inactive’ depending on whether the compound was predicted to interact or not with the target, ‘empty’ if the model was unable to make a prediction, or ‘both’ if it could not make a conclusion.

## 5. Conclusions

This investigation significantly advances our understanding of the promising therapeutic implications of pentacyclic triterpenoids in cancer treatment. By dissecting the structural differences associated with the regulation of cellular processes, we lay the groundwork for the strategic development of triterpene derivatives with superior efficacy. For instance, the basic structure of BeA provides a promising scaffold for the synthesis of novel lung cancer therapies, where substituent manipulations can be explored to enhance its activity. Similarly, OleA emerges as a focal point in unraveling the structural specificities that govern its selective effects on cancer cells. Our findings suggest opportunities for future research to delve deeper into the regulatory mechanisms influencing chemoresistance in these triterpenes. Towards clinical translation, it is imperative to develop derivatives that integrate the structural patterns of OleA and BeA to position them as robust future candidates.

## Figures and Tables

**Table 1:** IC_50_ and TI in A549 and MRC5 cells for each pentacyclic triterpene under study.

Triterpene	A549 IC_50_ (µM)	MRC5 IC_50_ (µM)	TI ^#^
AsA	59 ± 6 *	38 ± 2	0.64
OleA	43 ± 5 *	86 ± 2	2.00
UrA	23 ± 1 *	13 ± 1	0.57
BeA	15 ± 0.7 *	19 ± 0.4	1.27
Lupe	80 ± 6 *	101 ± 8	1.26
Betu	22 ± 1	16 ± 0.5	0.73

* These results have already been obtained by the authors [10]. ^#^ TI = MRC5 IC_50_/A549 IC_50_, where >1 means higher selectivity.

**Table 2:** Cancer-related targets that are theoretically predicted to interact with the six pentacyclic triterpenes.

**BeA Target** **ID: CHEMBL269277**	**Confidence 70, 80, 90%**	**Activity Threshold**	**OleA Target** **ID: CHEMBL168**	**Confidence 70, 80, 90%**	**Activity Threshold**
Interleukin-2	active	6	Interleukin-2	active	6
TNF-alpha	active	6	TNF-alpha	active	6
Dihydrofolate reductase	active	6	Dihydrofolate reductase	active	6
Adenosine deaminase	active	6	WD repeat-containing protein 5	active	6
WD repeat-containing protein 5	active	6			
LSD1/CoREST complex	active	6			
**Lupe Target** **ID: CHEMBL289191**	**Confidence 70, 80, 90%**	**Activity Threshold**	**UrA Target** **ID: CHEMBL169**	**Confidence 70, 80, 90%**	**Activity Threshold**
Interleukin-2	active	6	Interleukin-2	active	6
Dihydrofolate reductase	active	6	TNF-alpha	active	6
Adenosine deaminase	active	6	Dihydrofolate reductase	active	6
WD repeat-containing protein 5	active	6	WD repeat-containing protein 5	active	6
LSD1/CoREST complex	active	6			
Carbonic anhydrase IX	active	6			
**Betu Target** **ID: CHEMBL23236**	**Confidence 70, 80, 90%**	**Activity Threshold**	**AsA Target** **ID: CHEMBL404313**	**Confidence 70, 80, 90%**	**Activity Threshold**
Interleukin-2	active	6	Interleukin-2	active	6
Dihydrofolate reductase	active	6	TNF-alpha	active	6
Adenosine deaminase	active	6	LSD1/CoREST complex	active	6
WD repeat-containing protein 5	active	6	Mucosa-associated lymphoid tissue lymphoma translocation protein 1	active	6
LSD1/CoREST complex	active	6	Gamma-secretase	active	5

Matching colors are used to show similarities between the six triterpenes. Molecules in black show unique targets among the six triterpenes.

**Table 3:** Summary of the six pentacyclic triterpenes’ results against NSCLC A549 cells.

Mechanism	UrA	AsA	OleA	Lupe	BeA	Betu	Highest	Lowest
Cytotoxicity	**✔**	**✔**	**✔**	**✔**	**✔**	**✔**	BeA	Lupe
TI > 1	**-**	**-**	**✔**	**✔**	**✔**	**-**	OleA	UrA
Early apoptosis	**✔**	**✔**	**✔**	**✔**	**✔**	**✔**	UrA	Lupe
Caspase 3 activation	**✔**	**✔**	**✔**	**-**	**✔**	**✔**	UrA	Lupe
Cell cycle arrest	**✔**	**✔**	**✔**	**✔**	**✔**	**✔**	Betu	UrA
DNA damage	**✔**	**✔**	**-**	**-**	**-**	**-**	UrA	OleA
RRM1 downregulation	**✔**	**✔**	**-**	**✔**	**✔**	**✔**	BeA	OleA
Total ROS production	**✔**	**✔**	**✔**	**✔**	**✔**	**✔**	BeA	UrA
mROS production	**✔**	**✔**	**✔**	**✔**	**✔**	**✔**	BeA	Betu
Autophagy inhibition	**-**	**-**	**✔**	**✔**	**✔**	**-**	Lupe	Betu
MAPK/PI3K inhibition	**-**	**-**	**-**	**-**	**-**	**✔**	Betu	UrA
PDL1 downregulation	**✔**	**✔**	**✔**	**✔**	**✔**	**-**	BeA	Betu
STAT3 downregulation	**✔**	**✔**	**-**	**✔**	**✔**	**✔**	BeA	OleA

“✔” means induction and “**-**“ means non-induction of any of the tested processes.

## Data Availability

Data are available upon reasonable request.

## Supplementary Materials

The following supporting information can be downloaded at: https://www.mdpi.com/article/10.3390/ph17060694/s1, Figure S1: Confocal microscopy images of the A549 cells (2 μm) treated with the six triterpenes. Table S1. Whole set of molecular targets which are predicted to interact with the six pentacyclic triterpenes generated using ChEMBL.
