# Supplementary material for: Unraveling the Impact of Six Pentacyclic Triterpenes Regulating Metabolic Pathways on Lung Carcinoma Cells
Source: Pharmaceuticals (Basel). 2024 May 28;17(6):694. doi: 10.3390/ph17060694 (PMC11206507; doi:10.3390/ph17060694)
Supplement: Supplementary file 1 [file pharmaceuticals-17-00694-s001.zip › pharmaceuticals-2946721-supplementary.pdf]

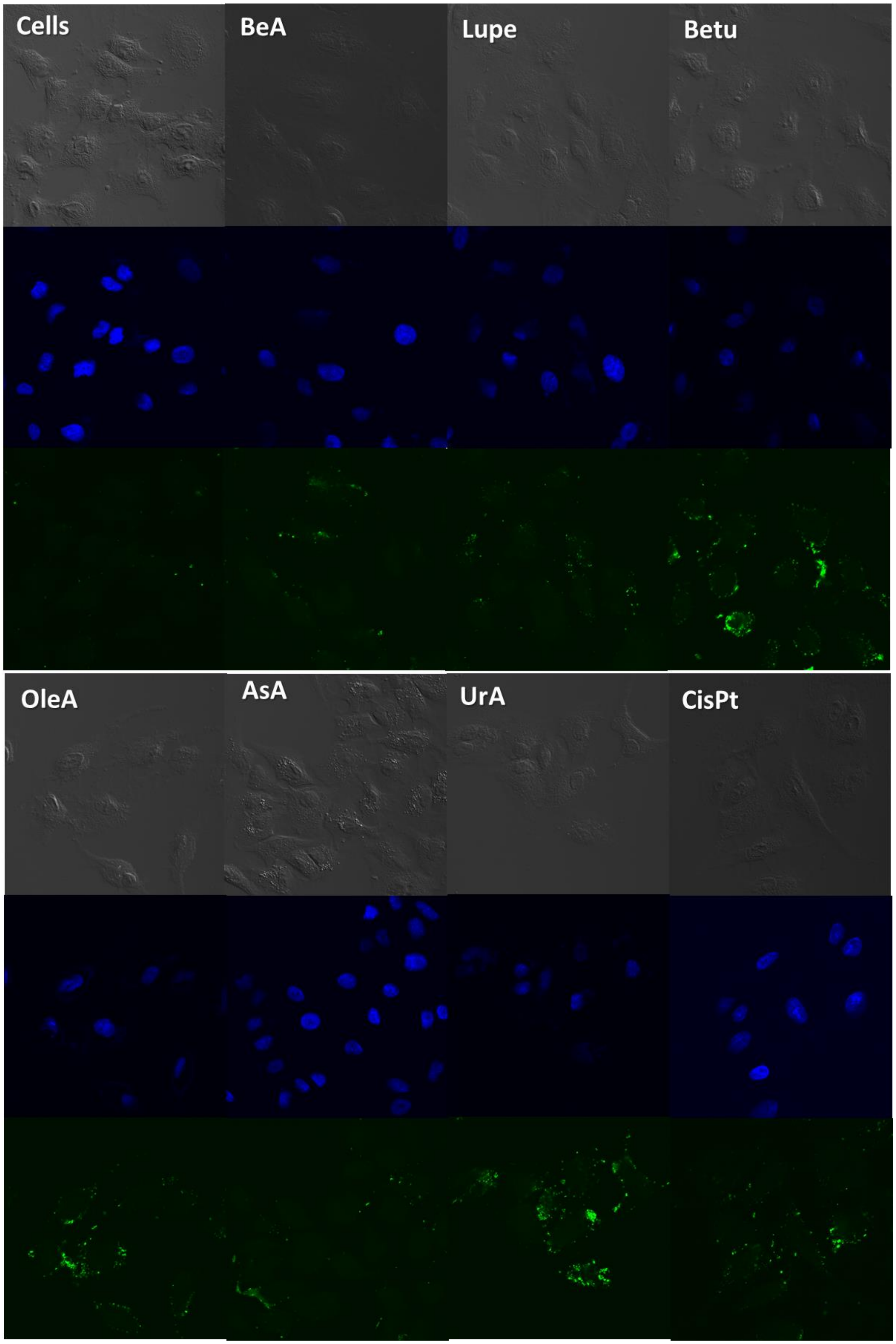

Figure S1. Confocal microscopy images of the A549 cells (2μm) treated with six triterpenes. These images show the following individual channels: Brightfield, DAPI (Ex/Em = 405/460nm), Annexin V Alexa Fluor (Ex/Em= 488/525 nm)

Table S1. Whole set of molecular targets which are predicted to interact with the six pentacyclic triterpenes generated using ChEMBL

Lupe Target ID: CHEMBL289191	Confidence 70, 80, 90%	Activity Threshold
Interleukin-2	active	6
Voltage-gated N-type calcium channel alpha-1B subunit	active	5
Sodium channel protein type III alpha subunit	active	5
Dihydrofolate reductase	active	6
Adenosine deaminase	active	6
Sodium/hydrogen exchanger 1	active	6
Monoglyceride lipase	active	6
WD repeat-containing protein 5	active	6
Glutamate NMDA receptor; GRIN1/GRIN2A	active	5
Peptidyl-prolyl cis-trans isomerase NIMA-interacting 1	active	6
LSD1/CoREST complex	active	6
Neuronal acetylcholine receptor; alpha3/beta4	active	5
Carbonic anhydrase IX	active	6

OleA Target ID: CHEMBL168	Confidence 70, 80, 90%	Activity Threshold
Interleukin-2	active	6
TNF-alpha	active	6
Voltage-gated N-type calcium channel alpha-1B subunit	active	5
Sodium channel protein type III alpha subunit	active	5
Dihydrofolate reductase	active	6
Phosphodiesterase 5A	active	6
Monoglyceride lipase	active	6
WD repeat-containing protein 5	active	6
Glutamate NMDA receptor; GRIN1/GRIN2A	active	5

AsA Target ID: CHEMBL404313	Confidence 70, 80, 90%	Activity Threshold
Interleukin-2	active	6
TNF-alpha	active	6
Sodium channel protein type III alpha subunit	active	5
Phosphodiesterase 5A	active	6
Monoglyceride lipase	active	6
Voltage-gated L-type calcium channel alpha-1C subunit	active	5
LSD1/CoREST complex	active	6
Mucosa-associated lymphoid tissue lymphoma translocation protein 1	active	6
Gamma-secretase	active	5

Matching colors are used to show similarities between the six triterpenes. Molecules in black show unique targets among the six triterpenes.

BeA Target ID: CHEMBL269277	Confidence 70, 80, 90%	Activity Threshold
Interleukin-2	active	6
TNF-alpha	active	6
Voltage-gated N-type calcium channel alpha-1B subunit	active	5
Sodium channel protein type III alpha subunit	active	5
Adenosine deaminase	active	6
Sodium/hydrogen exchanger 1	active	6
Dihydrofolate reductase	active	6
Monoglyceride lipase	active	6
WD repeat-containing protein 5	active	6
LSD1/CoREST complex	active	6
Glutamate NMDA receptor; GRIN1/GRIN2A	active	5

Betu Target ID: CHEMBL23236	Confidence 70, 80, 90%	Activity Threshold
Interleukin-2	active	6
Voltage-gated N-type calcium channel alpha-1B subunit	active	5
Sodium channel protein type III alpha subunit	active	5
Dihydrofolate reductase	active	6
Adenosine deaminase	active	6
Sodium/hydrogen exchanger 1	active	6
WD repeat-containing protein 5	active	6
Glutamate NMDA receptor; GRIN1/GRIN2A	active	5
Peptidyl-prolyl cis-trans isomerase NIMA-interacting 1	active	6
LSD1/CoREST complex	active	6

UrA Target ID: CHEMBL169	Confidence 70, 80, 90%	Activity Threshold
Interleukin-2	active	6
TNF-alpha	active	6
Voltage-gated N-type calcium channel alpha-1B subunit	active	5
Sodium channel protein type III alpha subunit	active	5
Dihydrofolate reductase	active	6
Phosphodiesterase 5A	active	6
Monoglyceride lipase	active	6
WD repeat-containing protein 5	active	6
Voltage-gated L-type calcium channel alpha-1C subunit	active	5
Glutamate NMDA receptor; GRIN1/GRIN2A	active	5

Grouping of the targets based on their cellular type and/or cellular organelle location

Membrane-associated proteins, receptors and ion channels:
Sodium channel protein type III alpha subunit
Carbonic anhydrase IX
WD repeat-containing protein 5 (WDR5)
Neuronal acetylcholine receptor; alpha3/beta4
Glutamate NMDA receptor; GRIN1/GRIN2A
Sodium/hydrogen exchanger 1 (NHE1)

Nuclear proteins:
LSD1/CoREST complex
Peptidyl-prolyl cis-trans isomerase NIMA-interacting 1 (PIN1)
WD repeat-containing protein 5 (WDR5)

Cytoplasmic enzymes:
Dihydrofolate reductase (DHFR)
Adenosine deaminase (ADA)
Monoglyceride lipase (MGLL)

Cytokines and signaling molecules:
TNF-alpha (Tumor Necrosis Factor-alpha)
Interleukin-2 (IL-2)
